# Comparison of the methylglyoxal scavenging effects of kaempferol and glutathione and the consequences for the toxicity of methylglyoxal in SH-SY5Y cells

**DOI:** 10.1016/j.fochx.2023.100920

**Published:** 2023-10-06

**Authors:** Liang Zheng, Wouter Bakker, Ignacio Miro Estruch, Frances Widjaja, Ivonne M.C.M. Rietjens

**Affiliations:** Division of Toxicology, Wageningen University and Research, Stippeneng 4, 6708 WE Wageningen, The Netherlands

**Keywords:** Methylglyoxal, Kaempferol, Glutathione, Adduct formation, Cytotoxicity

## Abstract

This study aimed to characterize the methylglyoxal (MGO) scavenging capacity of glutathione (GSH) and kaempferol in more detail with special emphasis on the possible reversible nature of the adduct formation and their competition for MGO, and the safety consequences of their MGO-scavenging effects. GSH showed immediate and concentration-dependent MGO-scavenging effects, while the scavenging effects by kaempferol appeared concentration- but also time-dependent, with stable adducts formed over time. The GSH adduct gradually disappeared in a competition reaction with kaempferol, and kaempferol became the preferred scavenger over time. Furthermore, the scavenging of MGO by kaempferol provided better protection than GSH against extracellular MGO in SH-SY5Y cells. It is concluded that flavonoids like kaempferol provide better scavengers for food-borne MGO than thiol-based scavengers such as GSH, while, given the endogenous concentrations of both scavengers and the detoxification of the GSH-MGO adduct by the glyoxalase system, GSH will be dominant for intracellular MGO protection.

## Highlights

• GSH showed immediate and concentration-dependent scavenging effects towards MGO, while the scavenging effects by kaempferol were concentration- but also time-dependent.
• The kaempferol adduct with MGO was stable over time, unlike the reversible adduct formed between GSH and MGO.
• The GSH adduct gradually disappeared in a competition reaction with kaempferol, and kaempferol became the preferred scavenger over time.
• The scavenging of MGO by kaempferol significantly decreased MGO-induced cytotoxicity and provided better protection than GSH against extracellular MGO.

## Graphical abstract

**Figure:**
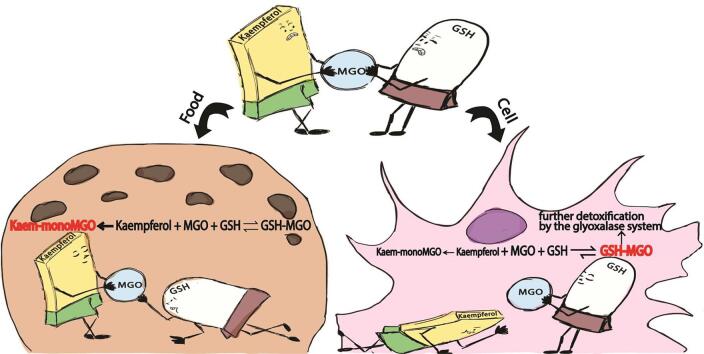

## 1 Introduction

Methylglyoxal (MGO) is a highly reactive α-dicarbonyl compound formed endogenous but also widely distributed in various foods and beverages such as roasted meat, cookies, bread, honey, coffee, milk, and beer (Degen et al., 2012, Maasen et al., 2021). It can be formed during food processing and storage due to the autoxidation of sugars, the Maillard reaction, lipid degradation, and/or enzymatic reactions of microorganisms in fermented foods (Hellwig, Gensberger-Reigl, Henle, & Pischetsrieder, 2018; Zheng et al., 2021). MGO plays an essential role in the color and aroma formation during thermal processing of some baked and fried foods (Nowotny, Schroter, Schreiner, & Grune, 2018). However, MGO can react with nucleophilic sites on proteins to form advanced glycation end products (AGEs), which may result in reduced nutritional value of foods (Zhu, Poojary, Andersen, & Lund, 2020b). Moreover, increasing evidence has indicated that higher intake of dietary AGEs may promote oxidative stress and inflammation, contributing to the development of some diseases such as chronic kidney disease and neurodegenerative diseases (Clarke et al., 2016, Garay-Sevilla et al., 2021). Considering the possible impact of MGO on food quality and human health, controlling the amount of MGO in foods is of importance, and the scavenging of MGO by different agents in foods has been suggested as a potential strategy to prevent the formation of AGEs and their adverse effects (Ghassem Zadeh and Yaylayan, 2021, Hamzalioglu and Gokmen, 2016, Li et al., 2014).

To this end, previous studies have reported the reactions of MGO with a variety of compounds, of which amino acids, thiol compounds, and plant polyphenols, especially flavonoids, have been proven to be efficient scavengers for MGO (Comert and Gokmen, 2019, Navarro and Morales, 2015). For instance, MGO reacts with amino groups of lysine to form reversible hemiaminals and stable AGEs including Nε-(carboxyethyl)lysine (CEL) and methylglyoxal-lysine dimer (MOLD) (Ahmed, Frye, Degenhardt, Thorpe, & Baynes, 1997), while the reaction with guanidine groups in arginine residues results in the irreversible formation of hydroimidazolones and argpyrimidines (Henle et al., 1994, Oya et al., 1999). MGO can also react reversibly with the thiol group of *N*-acetylcysteine and glutathione (GSH) to form hemithioacetals (Lo, Westwood, McLellan, Selwood, & Thornalley, 1994). Especially, the hemithioacetals formed by GSH and MGO can be further detoxified into d-lactate by glyoxalase enzymes in biological systems (Lo et al., 1994, Rae et al., 1990). This GSH conjugation is a well-known phenomenon identified before as a primary endogenous detoxification mechanism for MGO (Thornalley, 1993). Compared to some free amino acids such as lysine, arginine, and histidine, which may react with MGO to produce AGEs, the addition of biological thiols in foods may be a reasonable choice to be used as the competitive target for MGO inhibiting the formation of AGEs. Lastly, natural polyphenols, especially flavonoids, have been regarded as efficient scavengers of MGO and suggested for MGO control in food systems such as milk and cookies, provided their solubility and background flavor would not hamper this use (Lund and Ray, 2017, Ou et al., 2018, Zhu et al., 2019, Zhu et al., 2020a, Zhu et al., 2020b). Studies have shown that flavonoids such as quercetin and genistein can trap MGO by forming mono- and di-MGO adducts under physiological conditions (Li et al., 2014, Lv et al., 2011). Although the MGO scavenging capacity of flavonoids is extensively studied in in-vitro cell-free systems, the extent to which the resulting adduct formation is reversible and/or is preferred over a reaction with thiol compounds such as GSH remains to be established. In addition, to what extent the scavenging effects of both GSH and flavonoids contribute to the detoxification of food-borne exogenous MGO is still unknown.

In the present study, a representative thiol compound GSH, and kaempferol, a typical flavonoid widely present in fruits and vegetables, were chosen as model compounds. The study aimed to characterize their MGO scavenging capacity in more detail with special emphasis on the possible reversible nature of the adduct formation and their potential competition for MGO, as well as the consequences of their MGO-scavenging effects both endogenously and in food for MGO-induced cytotoxicity. To this end, product formation in in-vitro incubations of MGO with GSH and/or kaempferol, and incubations of purified adducts formed were characterized in time using an LC-MS based method. Furthermore, the influence of MGO scavenging by GSH and kaempferol on MGO-induced cytotoxicity was evaluated, using the SH-SY5Y human neuroblastoma cells, representing neural cells known to be sensitive towards dicarbonyl stress and/or AGEs (de Arriba et al., 2006, Nasu et al., 2020).

## 2 Materials and methods

### 2.1 Chemicals and reagents

MGO (40 % in water), l-glutathione reduced (GSH, ≥ 98 %), l-glutathione oxidized (GSSG, ≥ 98 %), 3-(4,5-dimethylthiazol-2-yl)-2,5-diphenyltetrazolium bromide (MTT, 98 %), dimethyl sulfoxide (DMSO, ≥ 99 %), ethanol (≥99 %), and acetic acid (≥99 %) were purchased from Merck (Darmstadt, Germany). Kaempferol (>99 %) was purchased from MedChemExpress (Monmouth Junction, NJ, USA). Acetonitrile (LC-MS grade) and methanol (LC-MS grade) were purchased from Biosolve BV (Valkenswaard, The Netherlands). Formic acid (≥99 %) was obtained from VWR CHEMICA (Amsterdam, The Netherlands). Dulbecco's Modified Eagle Medium/Nutrient Mixture F-12 with GlutaMAX supplement cell culture medium (DMEM/F12 GlutaMAX), penicillin/streptomycin, phosphate buffered saline (PBS), Hanks' balanced salt solution (HBSS), trypsin-EDTA and nonessential amino acids (NEAA) were bought from Gibco (Paisley, UK). Foetal calf serum (FCS) was purchased from Bodinco (Alkmaar, The Netherlands). Ultrapure water was prepared by a Milli-Q system (Millipore, MA, USA).

### 2.2 Kinetic study of the reaction between GSH and kaempferol with MGO

Stock solutions of MGO, GSH, and GSSG were prepared (5 mM) in 100  mM sodium phosphate buffer (pH = 7.4), and a stock solution of kaempferol (5 mM) was prepared in DMSO. To a starting solution of GSH or kaempferol (final concentration 0.25 mM) in 100 mM phosphate buffer (pH = 7.4, with 1.5 % DMSO for each incubation) were added different volumes of MGO stock solution to give a final concentration of 0.5, 1.25, or 2.5 mM (GSH or kaempferol, each with MGO in the molar ratios of scavenger: MGO of 1:2, 1:5, and 1:10). The resulting solutions were incubated at 37 °C in a water bath for 0, 1, 2, 4, 8, 24, and 48 h. At each time point, 100 μL stop solution (ethanol containing 2 % acetic acid) was added to the equal volume of collected samples to stop the reaction. Samples were immediately stored at −80 °C until analysis. LC-TOF-MS was applied for the analysis of kaempferol and its adducts with MGO as further described in section 2.5, and LC-TQ-MS was used for the quantification of GSH, GSSG, and the GSH adduct with MGO as described in section 2.6.

### 2.3 Reversibility of the MGO-kaempferol adducts

The purified monoMGO adduct of kaempferol was incubated (final concentration 5 μM) in 100 mM phosphate buffer (pH = 7.4) at 37 °C in a water bath for 24 h. After the same sampling step as applied in section 2.2, the samples were stored at −80 °C until analysis for free kaempferol by LC-TQ-MS as described in section 2.6.

### 2.4 Investigation of the competition between GSH and kaempferol for MGO adduct formation

To a starting solution of GSH and kaempferol (final concentration of 0.25 mM) in 100 mM phosphate buffer (pH = 7.4, with 1.5 % DMSO in the incubation) was added MGO stock solution to a final concentration of 0.25 mM. The resulting solutions were incubated at 37 °C in a water bath for 0, 1, 2, 4, 8, 24, and 48 h. Samples were collected in the same way as described in section 2.2, after which they were immediately stored at −80 °C until further analysis for kaempferol, GSH, GSSG, and the adducts formed between GSH and kaempferol with MGO by LC-TQ-MS as described in section 2.6.

### 2.5 LC-TOF-MS analysis

An Agilent 1200 LC system coupled with a Bruker micro-TOF mass spectrometer was used to analyze kaempferol and its MGO conjugates in the incubation mixtures of kaempferol with MGO, which enabled the identification and quantification of the diverse kaempferol adducts with MGO. An Acquity UPLC BEH C18 (50 mm × 2.1 mm, 1.7 µm) column was employed during the experiment with a flow rate of 0.18 mL/min. The mobile phase was composed of A (ultrapure water with 0.1 % formic acid) and B (acetonitrile with 0.1 % formic acid) with the following gradient: 0–5 min, 100–65 %A; 5–15 min, 65–40 %A; 15–18 min, 40–20 %A; 18–19 min, 20–100 %A; 19–30 min, 100 %A. Mass spectrometric analysis was performed in the negative electrospray ionization mode with mass spectra acquired from *m*/*z* 100 to 1500. The instrument parameters were: capillary voltage, + 3200 V; nebulizing gas pressure, 2 bar; drying gas flow, 8 L/min and drying temperature, 200 °C.

### 2.6 LC-TQ-MS analysis

A Shimadzu Nexera XR LC-20AD XR UHPLC system coupled with a Shimadzu 8050 triple quadrupole mass spectrometer with electrospray ionization (ESI) interface was applied for the kinetic study of the reaction between GSH and MGO by quantification of GSH, GSSG, and the GSH-MGO adduct. The *m*/*z* ratio of the GSH-MGO adduct for the LC-TQ-MS method was obtained by LC-TOF-MS in our previous study (Zheng, van Dongen, Bakker, Miro Estruch, & Rietjens, 2022). LC-TQ-MS methods were also developed for the quantification of kaempferol and its reaction products with MGO based on the identification information obtained by LC-TOF-MS in the current study, which also enabled simultaneous quantification of GSH, kaempferol, and the reaction products in a co-incubation of GSH and kaempferol with MGO. Chromatographic separation was achieved using a Supelco Discovery HS F5-3 column (15 cm × 2.1 mm, 3 μm). Ultrapure water containing 0.1 % formic acid (A) and acetonitrile with 0.1 % formic acid (B) were used as mobile phase at a flow rate of 0.25 mL/min. The following gradient was used: 0–2 min, 100 %A; 2–5 min, 100–40 %A; 5–11 min, 40 %–5%A; 11–14 min, 5 %A; 14–14.1 min, 5 %–100 %A; 14.1–24 min, 100 %A. The instrument parameters were as follows: nebulizing gas flow, 3.0 L/min; drying gas flow and heating gas flow, 10.0 L/min; interface temperature, 300 °C; and heat block temperature, 400 °C. Multiple-reaction monitoring (MRM) and selected-ion monitoring (SIM) modes with ionization polarity switching were utilized for the simultaneous determination of GSH, kaempferol, and the reaction products. The optimized acquisition parameters for these compounds are summarized in Table S1 (Supplementary file).

### 2.7 Cell culture

The SH-SY5Y (ATCC CRL-2266) human neuroblastoma cell line was purchased from the American Type Culture Collection (ATCC, Manassas, VA, USA). The cells were cultured in DMEM/F12 GlutaMAX medium containing 10 % FCS, 1 % NEAA, and 1 % penicillin/streptomycin in a humidified incubator at 5 % CO2 at 37 °C. Cells were subcultivated at a ratio of 1:20 upon confluency, using 0.05 % trypsin-EDTA solution. The medium was changed every 4 to 7 days.

### 2.8 Cell viability assay

To study the consequences of MGO scavenging by GSH and kaempferol for the toxicity of MGO, two types of experiments were performed. First, concentration–response curves were made for the cytotoxicity of MGO preincubated without or with a fixed concentration (0.25 mM) of GSH or kaempferol. Second, a fixed concentration (2 mM) of MGO was mixed with increasing concentrations of GSH (0.05–0.25 mM, final concentration) or kaempferol (0.05–0.25 mM, final concentration) either with or without subsequent pre-incubation after which cytotoxicity was quantified.

Pre-incubations were performed for 48 h at 37 °C in a cell-free HBSS system before exposure. The cell viability assay was performed using the MTT method. Briefly, SH-SY5Y cells (1.5 × 10^4^ cells/well) were seeded into 96-well plates (Greiner Bio-one) and incubated for 24 h. The culture medium was then replaced by the pre-incubated or non-preincubated solutions described above which were further supplemented with 10 % FCS. After 24 h exposure, the cells were carefully washed with HBSS and incubated for 3 h in fresh HBSS containing 10 % FCS and 0.5 mg/mL MTT. Subsequently, the produced formazan crystals were dissolved in 100 μL DMSO, after which the absorbance at 562 nm and at a reference wavelength of 620 nm was measured using a plate spectrophotometer (Molecular Devices, San Jose, CA, USA, Spectra Max M2). Corrected absorbance values were calculated by subtracting the values at 620 nm from the corresponding values at 562 nm. The cell viability was further expressed as a percentage relative to solvent control set at 100 %.

### 2.9 Statistical analysis

Data are expressed as mean ± standard error of the mean (SEM) from at least three independent experiments. Statistical analyses and visualization of the results were conducted using GraphPad Prism 9 software (San Diego, CA, USA). EC50 values were determined by non-linear regression analysis, and statistical differences between the EC50 values were analyzed using student's *t*-tests. A two-way ANOVA was employed to assess the cytotoxic effects induced by exposure to different concentrations of MGO, under pre-incubation conditions without or with a fixed amount of GSH or kaempferol. A Dunnett’s post hoc test was performed to compare the differences between the experimental groups and the control group (MGO pre-incubated alone group). Additionally, the effects of pre-incubation and non-preincubation of a fixed amount of MGO with increasing concentrations of GSH or kaempferol on the cytotoxicity of MGO were assessed using a two-way ANOVA, followed by a Tukey post hoc test for pairwise comparisons between any two groups. Results with *p-*values less than 0.05 were considered statistically significant.

## 3 Results

### 3.1 Characterization of reaction products between kaempferol and MGO by LC-TOF-MS

Our previous study already confirmed that a GSH-MGO adduct is formed upon incubation of GSH and MGO in phosphate buffer (Zheng et al., 2022). In the current study, we qualitatively analyzed the reaction products between kaempferol and MGO by LC-TOF-MS. Nine major reaction products were detected after incubation of kaempferol (0.25 mM) with MGO (2.5 mM) for 48 h. The identification information for kaempferol and its MGO adducts is summarized in Table S2, and typical extracted ion chromatograms are shown in Fig. S1 (Supplementary file). The peak at 12.0 min (Fig. S1B) had the molecular ion *m*/*z* 357.1 [M – H]^–^, which was 72 mass units higher than the molecular ion of kaempferol *m*/*z* 285.1 [M – H]^–^, indicating this peak represented a mono-MGO conjugated kaempferol (denoted as kaem-monoMGO). The peaks at 11.1, 11.5, and 12.1 min (Fig. S1C) had identical molecular ions of *m*/*z* 429.1 [M – H]^–^ with 72 mass units higher than the *m*/*z* of the kaem-monoMGO adduct (*m*/*z* 357.1 [M – H]^–^) indicating that they were isomers of diMGO adducts of kaempferol (denoted as kaem-diMGOa, kaem-diMGOb, and kaem-diMGOc), with kaem-diMGOa being the dominant isomer. The molecular ions of the peaks at 12.0, 13.0, and 14.0 min (*m*/*z* 355.1 [M – H]^–^, Fig. S1D) were 2 mass units lower than that of the kaem-monoMGO (*m*/*z* 357.1 [M – H]^–^), indicating a possible loss of two protons after the formation of kaem-monoMGO, and thus these peaks were proposed as the isomers of oxidized kaem-monoMGO (denoted as oxidized kaem-monoMGOa, oxidized kaem-monoMGOb, and oxidized kaem-monoMGOc). Similarly, the peaks at 11.5 and 12.0 min (Fig. S1E) had molecular ions of *m*/*z* 427.1 [M – H]^–^ with 2 mass units lower than the *m*/*z* of diMGO adduct of kaempferol (*m*/*z* 429.1 [M – H]^–^), suggesting the loss of two protons after the conjugation of two molecules of MGO to kaempferol (denoted as oxidized kaem-diMGOa and oxidized kaem-diMGOb).

### 3.2 Kinetic study of the reaction between GSH and kaempferol with MGO

**Fig. 1:**
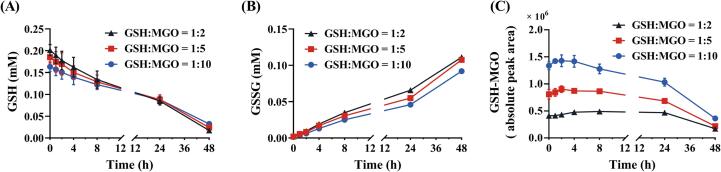
Time-dependent changes in the contents of GSH (A), GSSG (B), and GSH-MGO adduct (C) during incubation of GSH (0.25 mM) with MGO (0.5, 1.25, or 2.5 mM) at three different molar ratios of 1:2, 1:5, and 1:10 in 100 mM sodium phosphate buffer (pH = 7.4) at 37 °C. The contents for GSH and GSSG were expressed as molar concentrations, while the content for GSH-MGO was expressed as absolute peak area. Data are presented as the means ± SEM of three replications.

The kinetics of the scavenging effects of GSH on MGO were evaluated under three molar ratios of GSH: MGO (1:2, 1:5, and 1:10) keeping the concentration of GSH constant. LC-TQ-MS was applied for the targeted determination of GSH, GSSG, and the GSH-MGO adduct. Fig. 1 shows the time-dependent changes in the contents of GSH, GSSG, and the GSH-MGO adduct during the incubation of GSH with MGO for 48 h. The concentrations of GSH and GSSG were determined via calibration curves using their commercially available reference compounds, while the amount of GSH-MGO adduct was expressed as peak area due to a lack of reference compounds. As shown in Fig. 1A, an average of 19.4 % (0.05 mM), 26.0 % (0.07 mM), and 34.7 % (0.09 mM) loss of GSH occurred immediately at the start of incubation at the molar ratios of GSH: MGO of 1:2, 1:5, and 1:10, respectively. A further continuous decrease in GSH levels accompanied by an increase in GSSG content during the subsequent 48 h was observed likely mainly due to the autooxidation of GSH to GSSG (Fig. 1A and B). The content of GSH-MGO adducts detected in the samples showed a slight tendency to increase in the first few hours followed by a decrease, the latter potentially related to the reversible nature of the adduct formation combined with the autooxidation of GSH (Fig. 1C). The amount of GSH-MGO adduct detected at the start of the incubations appeared to vary depending on the amount of MGO added, indicating the adduct formation to be instantaneous on the time scale used.

**Fig. 2:**
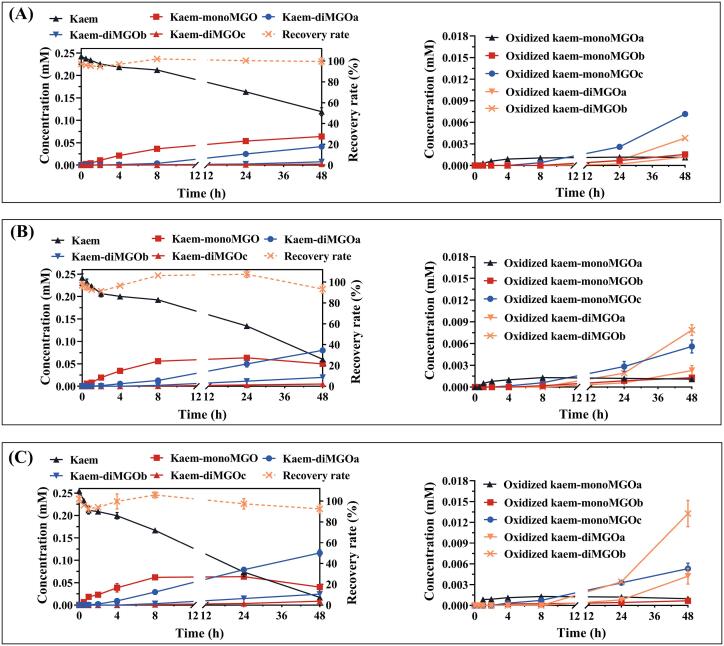
Time dependent changes in the concentrations of kaempferol (kaem), its reduced reaction products (left graph), and oxidized reaction products (right graph) after incubation of kaempferol (0.25 mM) with MGO (0.5, 1.25, or 2.5 mM) at three different molar ratios of 1:2 (A), 1:5 (B), and 1:10 (C) in 100  mM sodium phosphate buffer (pH = 7.4) at 37 °C. Data are presented as mean ± SEM of three replications.

The kinetics of MGO scavenging by kaempferol were evaluated under the same incubation conditions as what was used for the incubations of GSH with MGO. Due to the more diverse formation of the reaction products between kaempferol with MGO compared to what was formed during incubation of GSH with MGO, LC-TOF-MS in full-scan acquisition mode was used for the analysis of the samples which enabled the comprehensive detection and quantification of the different reaction products. Kaempferol was quantified using its reference compound, and its reaction products were quantified using kaempferol as the reference in extracted ion mode. Using this approach the total mass recoveries for kaempferol and its reaction products ranged from 91.2 % to 107.6 %. As shown in Fig. 2, the concentration of kaempferol decreased with increasing reaction time, with 47.7 %, 24.1 %, and 6.9 % of kaempferol remaining after 48 h of incubation at the molar ratios of kaempferol: MGO of 1:2, 1:5, and 1:10, respectively. The content of kaem-monoMGO increased rapidly with incubation time in the first eight hours at all three ratios, and a slight decrease was observed after 24 h for the ratios of 1:5 and 1:10. The levels of the diMGO adducts of kaempferol increased with increasing incubation time during the 48 h of incubation, with kaem-diMGOa being the most abundant. Besides, the oxidized reaction products were also found to be slowly formed during the entire incubation period. At the start of the incubations kaempferol appeared to be present in its unmodified form.

### 3.3 Reversibility of MGO adducts of kaempferol

**Fig. 3:**
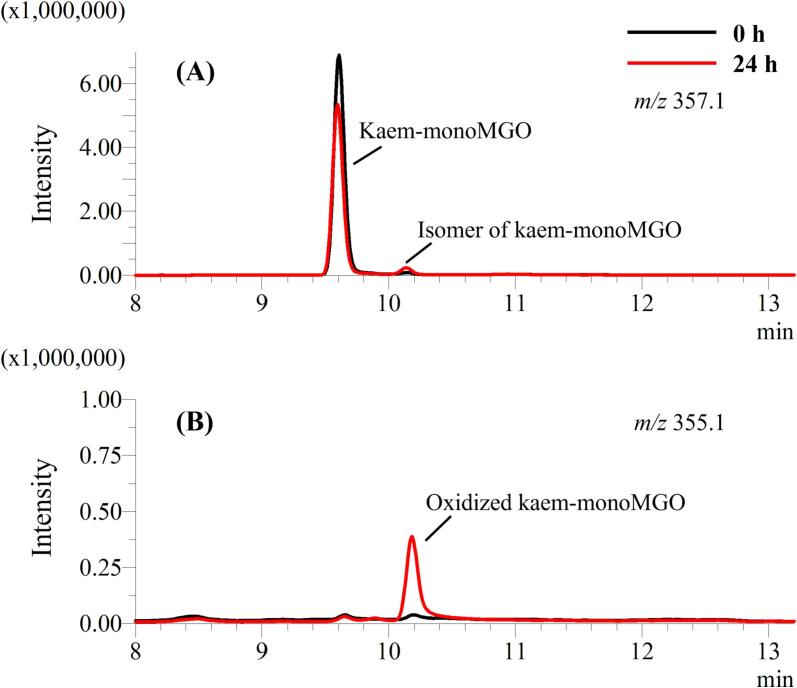
Typical LC-TQ-MS extracted ion chromatograms of kaempferol-monoMGO (A) and oxidized kaempferol-monoMGO (B) after incubation of kaempferol-monoMGO in 100 mM sodium phosphate buffer (pH = 7.4) at 37 °C for 0 and 24 h.

A previous study has reported the reversibility of the adduct formation between GSH with MGO (Andreeva et al., 2019). In this section of the study, we evaluated the reversibility of the adducts formed between kaempferol with MGO by incubation of kaem-monoMGO which was isolated and purified by preparative LC. The incubation mixtures were analyzed by LC-TQ-MS by monitoring the changes in the peak areas of kaem-monoMGO, its potential oxidized products, and kaempferol in SIM mode. Incubation of kaem-monoMGO for 24 h resulted in a decrease in its content by 22.3 %, while significant increases in the isomer of kaem-monoMGO and oxidized products were observed (Fig. 3A and B). In addition, the amount of kaempferol remained at < 0.8 % and no significant change in its content was found during and after 24 h incubation. This result indicates that the monoMGO adduct formation of kaempferol was not reversible and that the reduction in the monoMGO adduct after 24 h is likely due to its oxidation and the formation of the isomer.

### 3.4 Competition between GSH and kaempferol for MGO adduct formation

**Fig. 4:**
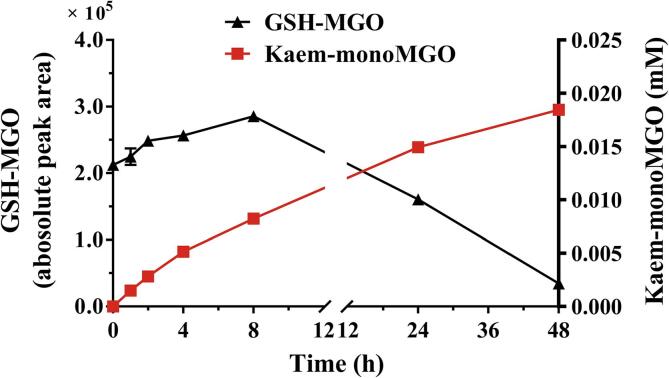
Changes in the contents of GSH-MGO adduct and kaem-monoMGO after incubation of GSH (0.25 mM) and kaempferol (0.25 mM) with MGO (0.25 mM) in 100 mM sodium phosphate buffer (pH = 7.4) at 37 °C for 48 h. The content of GSH-MGO was expressed as absolute peak area, and the content for kaem-monoMGO was expressed as molar concentration. Data are presented as mean ± SEM of three replications.

GSH and kaempferol were co-incubated with MGO to study the potential competition between GSH and kaempferol for MGO. Fig. 4 shows the changes in the contents of the GSH-MGO adduct and kaem-monoMGO (the main MGO adduct of kaempferol under this incubation condition) upon incubation of GSH and kaempferol with MGO for 48 h. Instantaneous formation of the GSH-MGO adduct at the start was observed. The content of the GSH-MGO adduct started to decrease after 8 h incubation, with 16.2 % of the GSH-MGO adduct remaining after 48 h relative to the starting point. However, the kaempferol-monoMGO adduct showed a constant increase in its concentration. The changes in the concentrations of GSH, GSSG, and kaempferol in this incubation of GSH and kaempferol with MGO can be found in Fig. S2. The GSH levels reduced by 17.9 % at the start followed by a rapid decrease (Fig. S2A). The reductions in GSH levels during the subsequent 48 h of the incubations were mainly due to the formation of GSSG from GSH (Fig. S2B). Fig. S2C shows that the concentration of kaempferol decreased gradually with increasing incubation time, with a 16.3 % decrease in kaempferol concentration after 48 h. The results show that kaempferol still exhibited time-dependent scavenging effects towards MGO in a co-incubation with GSH and MGO, and it can be expected that the remaining MGO would continually be scavenged by kaempferol over time, whereas the GSH-MGO would eventually be fully deconjugated with the resulting MGO being scavenged by kaempferol. As a result, in incubations of MGO with both GSH and kaempferol the adducts of MGO with kaempferol would become the preferred adducts over time due to their irreversible nature.

### 3.5 Influence of MGO scavenging by GSH and kaempferol on the cytotoxicity of MGO in SH-SY5Y cells

**Fig. 5:**
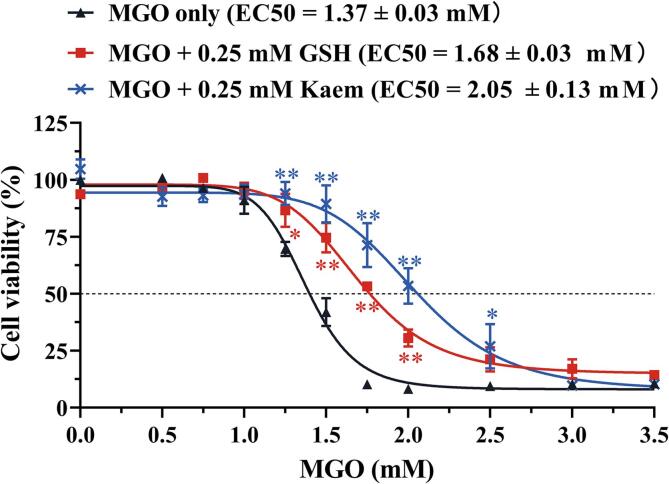
Effects of 48 h pre-incubation of MGO with GSH or kaempferol on the reduction of cell viability induced by increasing concentrations of MGO in SH-SY5Y cells as assessed by MTT assay. Data are presented as mean ± SEM of at least four replications. * *p* < 0.05 and ** *p* < 0.01 compared with the MGO pre-incubated alone group.

The effects of different concentrations of GSH (0.05–0.25  mM) and kaempferol (0.05–0.25 mM) on the cell viability of SH-SY5Y cells were evaluated by the MTT assay. Fig. S3 shows that GSH and kaempferol at all concentrations tested up to 0.25 mM did not affect the cell viability of SH-SY5Y cells. To assess the effects of the scavenging of MGO by GSH and kaempferol on the MGO-induced cytotoxicity, MGO at different concentrations was pre-incubated in the absence and presence of 0.25 mM GSH or kaempferol for 48 h before exposure of cells to the resulting incubation mixtures. The difference in cell viability between the exposed groups is shown in Fig. 5. Two-way ANOVA analysis of these data confirmed a significant effect of treatment (*p* < 0.001), concentration (*p* < 0.001), and their interaction (*p* < 0.001). Post hoc analysis using Dunnett’s test revealed that pre-incubation of MGO with GSH and kaempferol both resulted in protection against MGO-induced cytotoxicity, with cell viability being statistically significantly higher upon preincubation of MGO with both scavengers than what was observed for MGO alone. Pre-incubation of MGO with GSH or kaempferol resulted in a statistically significant (*p* < 0.01) increase in the EC50 values (1.68 and 2.05 mM, respectively) compared to the EC50 of MGO pre-incubated alone (1.37 mM). In addition, the mean EC50 value of MGO pre-incubated with 0.25 mM kaempferol was significantly higher than the EC50 value of MGO pre-incubated with 0.25 mM GSH (*p* < 0.05), indicating kaempferol to be a better scavenger under the conditions employed.

**Fig. 6:**
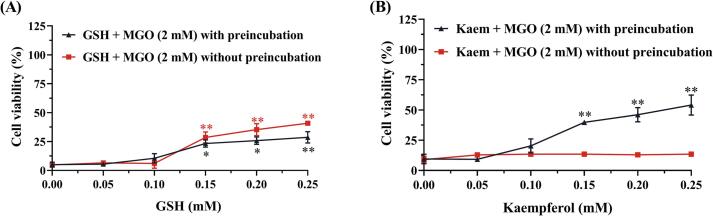
Comparison of the effects of 48 h pre-incubation and non-preincubation of MGO (2 mM) with increasing concentrations of GSH (A) or kaempferol (B) on the decrease of cell viability induced by MGO in SH-SY5Y cells as assessed by MTT assay. Data are all presented as mean ± SEM of at least three replications. * *p* < 0.05 and ** *p* < 0.01 compared with the MGO-only group.

The consequences of scavenging of MGO by GSH or kaempferol for MGO-induced cytotoxicity were further investigated. As shown in Fig. 6A, protection against MGO-induced cytotoxicity by GSH appeared independent of preincubation. Testing with or without pre-incubation did not significantly influence the protective effects of GSH against MGO-induced reduction in cell viability. In contrast, Fig. 6B shows that without pre-incubation kaempferol did not significantly influence the MGO-induced effects on cell viability, while the viability was significantly increased after 48 h pre-incubation of MGO with kaempferol at concentrations ≥ 0.15 mM. Thus, these results corroborate, in line with what was observed in the cell-free incubations, that scavenging of MGO by kaempferol is, unlike the scavenging by GSH, not instantaneous, and requires some preincubation to display the protective effect whereas, in line with the instantaneous nature of its reaction with MGO, scavenging by GSH was protective without a need for preincubation.

## 4 Discussion

The present study compared the MGO scavenging behavior of a representative thiol compound (GSH) and a typical dietary flavonoid (kaempferol) in more detail with special emphasis on the reversible nature of the adducts formed and the resulting potential for protection against MGO-induced cytotoxicity. The scavenging of MGO by GSH to form GSH-MGO hemithioacetals is a well-known phenomenon that occurs spontaneously and immediately and is known to be reversible leading to an equilibrium (Andreeva et al., 2019, Kammerscheit et al., 2020). The kinetic results in this study also indicate that an increase in the proportion of MGO relative to GSH (GSH: MGO = 1:2, 1:5, and 1:10) shifted the equilibrium towards the GSH-MGO adduct formation. In contrast, kaempferol presented a different scavenging behavior towards MGO. Results obtained show that kaempferol can form various adducts with MGO, including mono- and di-MGO adducts in both reduced and oxidized forms. The structures of reduced mono- and di-MGO adducts of various flavonoids have been previously reported (Li et al., 2014, Lv et al., 2011, Zhang et al., 2022). The C6 and C8 positions of the A ring in the flavonoids are identified as the trapping sites of MGO (Lv et al., 2011). In a study of the reaction between naringenin and MGO, C8 was reported to be the site with the highest reactivity due to its highest electron density among all the carbon atoms, and the mono-MGO adduct can be formed by reacting with one molecule of MGO at the C8 position followed by the formation of the di-MGO adduct by reacting at C6 with a second molecule of MGO (Zhu et al., 2019). The MGO adducts of flavonoids were found to exist in several isomeric forms. For example, Liu, Xia, Lu, Zheng, Sang, and Lv (2017) observed two isomers of the mono-MGO adduct and four isomers of the di-MGO adduct of quercetin by LC-MS/MS. A study by Zhu et al. (2020a) detected two mono-MGO adducts and one di-MGO adduct of kaempferol by LC-MS/MS, while one mono-MGO adduct and three di-MGO adducts of kaempferol were detected by LC-TOF-MS in the present study. The differences between these studies could be due to the different chromatographic conditions leading to different resolutions for separating the isomers. Besides, some isomers may be present in the solution at a concentration that is below the detection limit. This would also explain the detection of the other isomer of kaem-monoMGO when LC-TQ-MS, which is more sensitive than LC-TOF-MS, was applied for the detection of the reversibility of kaem-monoMGO. With respect to the oxidized MGO adducts of the flavonoids, studies on their structures are still limited. A recent study identified the structures of several oxidized di-MGO adducts of rutin (Chen et al., 2022). The oxidation was reported to occur at the hydroxy group in the MGO moiety after the formation of the di-MGO adduct of rutin (Chen et al., 2022). Besides, a study investigating the quercetin-MGO adducts in a lysine/glucose aqueous system, suggested that the oxidation of the adducts may occur at the hydroxy group of quercetin to form mono- and di-MGO adducts of quercetin quinone, while the exact oxidation sites on the quercetin moiety remained unknown (Liu et al., 2017). For oxidized MGO adducts of kaempferol, the oxidation in the kaempferol moiety may also be possible due to the presence of the hydroxyl moieties at the C3, C4′-position which are sensitive sites to oxidation (Moridani, Galati, & O'Brien, 2002). Based on the above, the proposed structures of the MGO adducts of kaempferol detected in this study are shown in Fig. S4. The kinetic results obtained show that the MGO-scavenging effect of kaempferol is time-dependent, and an increase in the MGO proportion in the cell-free incubations of MGO with kaempferol (kaempferol: MGO = 1:2, 1:5, and 1:10) resulted in a more rapid loss of kaempferol during 48 h with an increasing relative concentration of MGO.

A previous study quantified the apparent second order rate constants for the reaction between four selected polyphenols (including kaempferol) and MGO (Zhu et al., 2020a). The apparent second order rate constant for the reaction of MGO with kaempferol amounted to 6.3 × 10^-2^ M^−1^s^−1^. The second order rate constant for the reaction of MGO with *N*-acetylcysteine as a thiol scavenger was reported to amount to 4.1 × 10^4^ M^−1^s^−1^ (Lo et al., 1994). These data corroborate that the initial scavenging of MGO by a thiol reagent like *N*-acetylcysteine or GSH will be highly favored over that by kaempferol. However, the reversible nature of the thiol adduct formation with a reverse first order rate constant of 7.5 × 10^-3^ s^−1^ (Lo et al., 1994) explains why in the longer term the irreversible scavenging by kaempferol dominates over scavenging by GSH. These kinetic constants are in line with the observed adduct formation and competition between kaempferol and GSH observed in the present study.

We further evaluated and compared the consequences of the MGO-scavenging effects of GSH and kaempferol for the cytotoxicity of MGO. Pre-incubation of MGO with kaempferol for 48 h resulted in a higher protection against MGO-induced cytotoxicity than its pre-incubation with GSH. The results also show that pre-incubation is not needed for GSH to display protective effects due to its instantaneous reaction with MGO. In contrast, the protective effects of kaempferol against MGO-induced cytotoxicity were shown to be dependent on a pre-incubation time, which can be ascribed to the substantially lower second order rate constant (6.3 × 10^-2^ M^−1^s^−1^) for its reaction with MGO (Zhu et al., 2020a) combined with the irreversible nature of the adduct formed as shown in the present study. It is of interest to note that a previous study evaluated the toxicity of oxidized MGO rutin adducts (oxidation at the MGO moiety) in comparison with rutin and MGO and suggested that the formation of these oxidized adducts significantly decreased the MGO-induced cytotoxicity in several cell types (Chen et al., 2022). In contrast to this and also to our study results, another study reported that the mono-MGO and di-MGO adducts of quercetin were found to have higher cytotoxic effects than MGO itself (Liu et al., 2021). To what extent these differences are due to the biological effects of different types of adducts, the different flavonoids, or different experimental conditions used needs further investigation. In addition, it is of interest to note that some studies have reported that the protective effects of several polyphenols against MGO-induced toxicity are correlated to their activation of Nrf2-mediated protective gene expression (Cha et al., 2018, de Oliveira et al., 2019, Zhou et al., 2019). However, this may be related to a mode of action where pre-treatment of cells with these compounds for a few hours to a day results in an increase in intracellular GSH levels via the activation of the Nrf2-mediated pathway prior to MGO exposure, as intracellular GSH plays an essential role in the detoxification of MGO (Zheng et al., 2022).

Given that intracellular levels of GSH are generally high and in the mM range (Meister & Anderson, 1983), while physiological levels of flavonoids are generally in the low μM range (Erlund et al., 2000; Erlund, Meririnne, Alfthan, & Aro, 2001), it may be concluded that for protection against endogenous MGO, GSH will be more relevant. However, the results of the present study reveal that for scavenging food-borne MGO use of kaempferol and other flavonoids would be preferred over the use of GSH and other thiol-based scavengers, as the GSH adduct would eventually lose MGO in a competition reaction with kaempferol. Our study reveals that the scavenging of MGO by kaempferol is not reversible and that the MGO scavenging mediated by kaempferol would lead to the detoxification of MGO, while the protective effects of GSH against MGO-induced cytotoxicity via extracellular MGO scavenging were less substantial. This conclusion would also hold when considering scavenging food-borne MGO. When considering scavenging of intracellular MGO however, GSH would be more efficient than kaempferol, not only because of its higher intracellular concentrations but also because of the fact that within the cell the reversibility of the adduct formation will be less problematic given that the GSH-MGO adducts are further detoxified by the glyoxalase system (Rae et al., 1990). Thus, taking all together it is concluded that for scavenging food-borne MGO kaempferol would be preferred over thiol scavengers, while for endogenous protection against MGO, the role of scavenging by GSH will be dominant.

## CRediT authorship contribution statement

**Liang Zheng:** Formal analysis, Investigation, Methodology, Validation, Visualization, Writing – original draft. **Wouter Bakker:** Methodology, Writing – review & editing, Supervision. **Ignacio Miro Estruch:** Methodology, Writing – review & editing. **Frances Widjaja:** Formal analysis, Visualization, Writing – review & editing. **Ivonne M.C.M. Rietjens:** Conceptualization, Methodology, Project administration, Writing – review & editing, Supervision.

## Declaration of Competing Interest

The authors declare the following financial interests/personal relationships which may be considered as potential competing interests: Liang Zheng reports financial support was provided by China Scholarship Council.

## Data Availability

Data will be made available on request.
