# Supplementary material for: Comparison of the methylglyoxal scavenging effects of kaempferol and glutathione and the consequences for the toxicity of methylglyoxal in SH-SY5Y cells
Source: Food Chem X. 2023 Oct 6;20:100920. doi: 10.1016/j.fochx.2023.100920 (PMC10740108; doi:10.1016/j.fochx.2023.100920)
Supplement: Supplementary data 1 [file mmc1.pdf]

Supplementary Materials

Comparison of the methylglyoxal scavenging effects of kaempferol and glutathione and the consequences for the toxicity of methylglyoxal in SH-SY5Y cells

Liang Zheng*, Wouter Bakker, Ignacio Miro Estruch, Frances Widjaja, Ivonne M. C. M. Rietjens

Division of Toxicology, Wageningen University and Research, Stippeneng 4, 6708 WE Wageningen, The Netherlands

* Corresponding author: Liang Zheng, e-mail address: liang. zheng@wur.nl (L. Zheng).

Table S1. LC-TQ-MS acquisition parameters.

Compounds	Scan mode (Polarity)	Precursor ion (*m/z*)	Product ion (*m/z*)	Collision energy (V)
GSH	MRM (+)	308.0	179.1	-13
GSSG	MRM (–)	611.1	306.0, 143.1	24, 48
GSH-MGO adduct	MRM (+)	380.2	308.3, 162.0	-13, -26
Kaempferol (Kaem)	SIM (–)	285.1	/	/
	MRM (–)	285.1	257.2, 239.1	19, 27
Kaem-monoMGO	SIM (–)	357.1	/	/
	MRM (–)	357.1	285.1, 205.0, 151.0	35, 35, 35
Kaem-diMGO	SIM (–)	429.1	/	/
	MRM (–)	429.1	357.1, 295.0, 285.1	35, 35, 35
Oxidized kaem-monoMGO	SIM (–)	355.1	/	/
Oxidized kaem-diMGO	SIM (–)	427.1	/	/

Table S2. LC-TOF-MS identification of kaempferol and its reaction products with MGO

Category	Compounds	Precursor ion (*m/z*)	Retention time (min)
Parent compound	Kaempferol (Kaem)	285.1	13.6
Reduced products	Kaem-monoMGO	357.1	12.0
	Kaem-diMGOa	429.1	11.1
	Kaem-diMGOb	429.1	11.5
	Kaem-diMGOc	429.1	12.1
Oxidized products	Oxidized kaem-monoMGOa	355.1	12.0
	Oxidized kaem-monoMGOb	355.1	13.0
	Oxidized kaem-monoMGOc	355.1	14.0
	Oxidized kaem-diMGOa	427.1	11.5
	Oxidized kaem-diMGOb	427.1	12.0

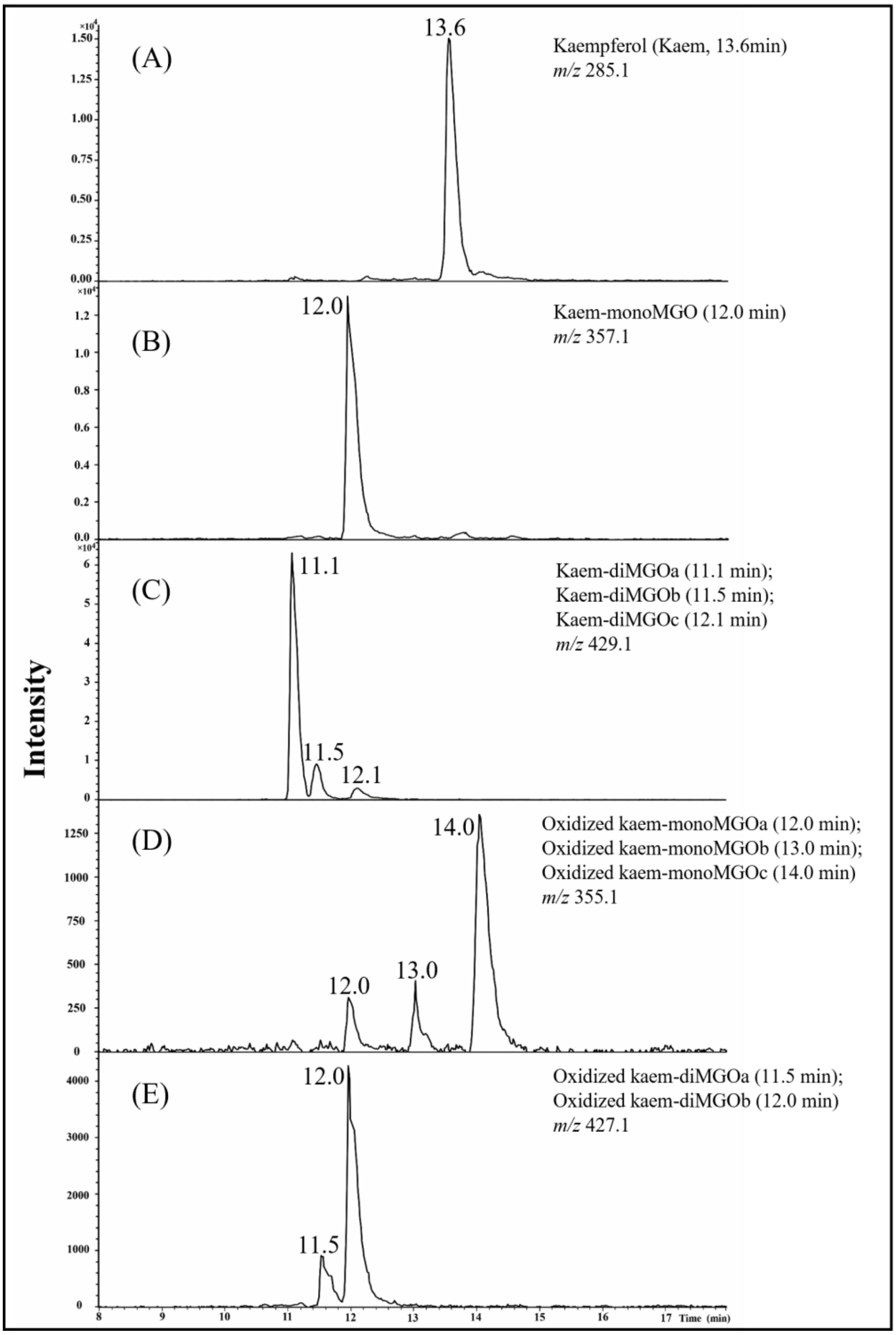

Fig. S1. Typical LC-TOF-MS extracted ion chromatograms of incubations of kaempferol (0.25 mM) with MGO (2.5 mM) for 48 h: (A) kaempferol (kaem, *m/z* = 285.1); (B) kaem-monoMGO (*m/z* = 357.1); (C) kaem-diMGOa, kaem-diMGOb; and kaem-diMGOc (*m/z* = 429.1); (D) oxidized kaem-monoMGOa, oxidized kaem-monoMGOb, and oxidized kaem-monoMGOc (*m/z* = 355.1); (E) oxidized kaem-diMGOa and oxidized kaem-diMGOb (*m/z* = 427.1).

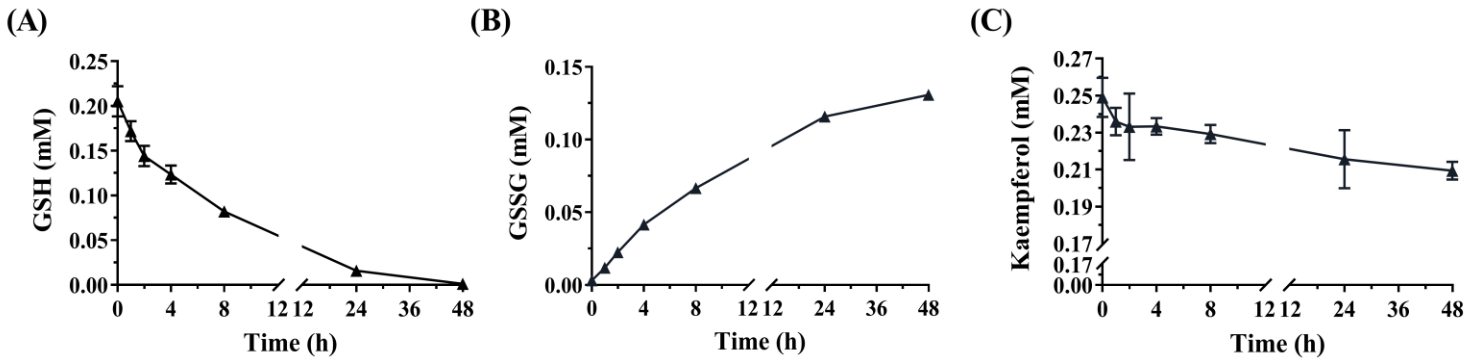

Fig. S2. Changes in the contents of GSH (A), GSSG (B), kaempferol (C) after incubation of GSH (0.25 mM) and kaempferol (0.25 mM) with MGO (0.25 mM) in 100 mM sodium phosphate buffer (pH=7.4) at 37 °C for 48 h. Data are presented as mean ± SEM of three replications.

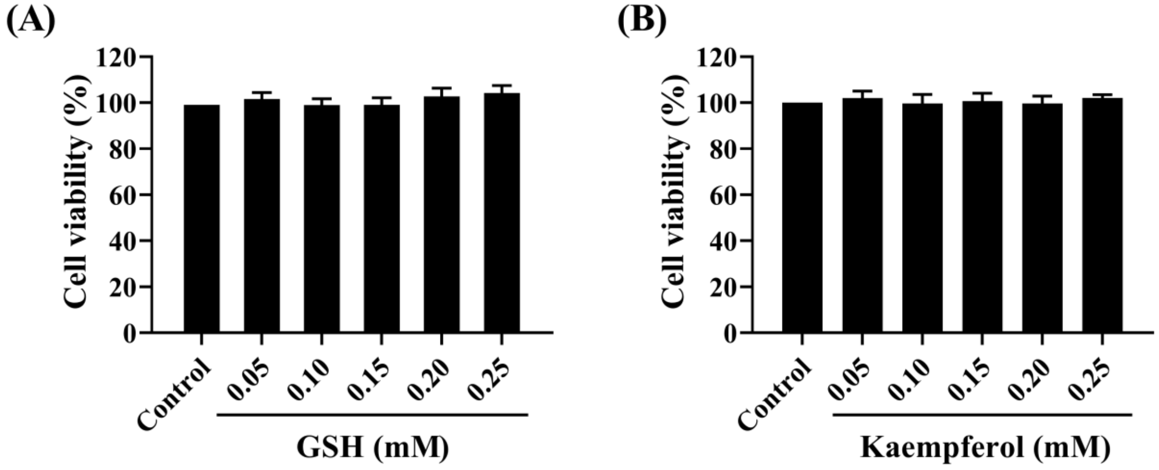

Fig. S3. Effects of GSH (A) and kaempferol (B) on the cell viability of SH-SY5Y cells. The results were calculated relative to solvent control and are presented as mean ± SEM of three replications.

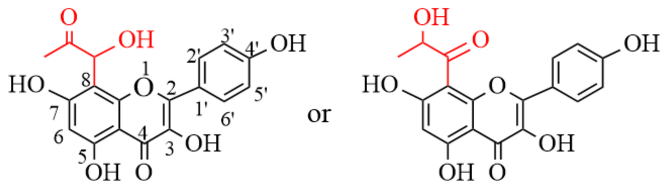

Kaem-monoMGO

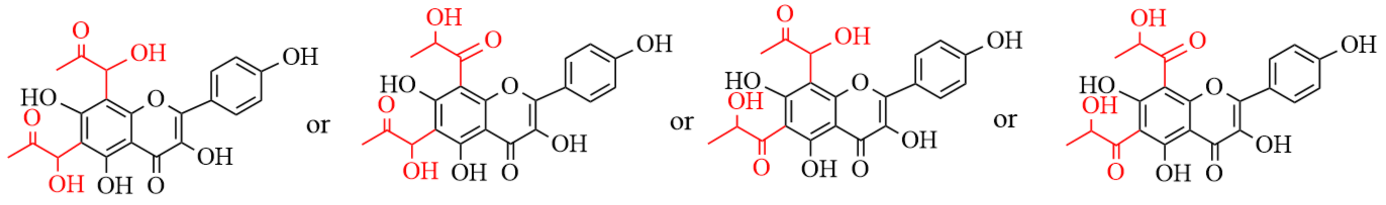

Kaem-diMGO

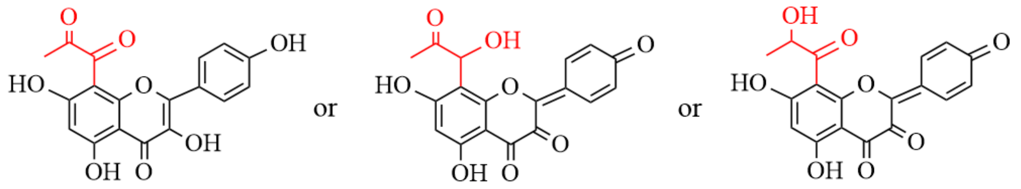

Oxidized kaem-monoMGO

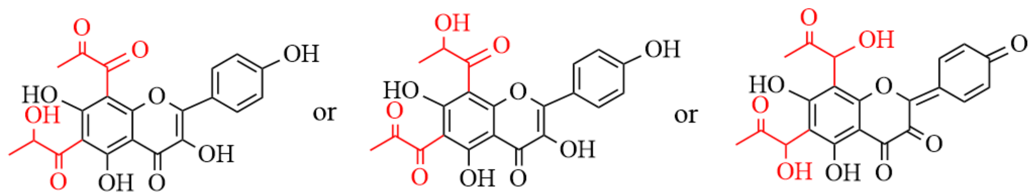

Oxidized kaem-diMGO

Fig. S4. Proposed structures of MGO adducts of kaempferol. Other isomeric forms for oxidized kaem-diMGO are also possible.
